# Internal Flow Fraction discriminates patients with dyssynchronous heart failure from age and sex-matched controls

**DOI:** 10.1186/1532-429X-11-S1-O92

**Published:** 2009-01-28

**Authors:** Brandon K Fornwalt, Jana G Delfino, Calvin R Kurz, Patrick C Gonzales, Robert Eisner, Angel R León, John N Oshinski

**Affiliations:** Emory University School of Medicine, Atlanta, GA USA

**Keywords:** Cardiac Resynchronization Therapy, Internal Flow, Mechanical Dyssynchrony, Left Ventricle Ejection Fraction, Left Ventricular Dyssynchrony

## Objective

Evaluate the ability of Internal Flow Fraction to diagnose left ventricular dyssynchrony using standard cine MRI.

## Background

Better methods to quantify mechanical dyssynchrony in the heart may improve patient selection for cardiac resynchronization therapy. Dyssynchrony creates inefficient "sloshing" of blood volume *internally* within the left ventricle (LV). This "internal flow" represents wasted energy due to the dyssynchronous motion of the LV walls. We developed a new method to quantify internal flow from cine cardiac MRI which may provide a better, more physiologic measure of dyssynchrony than existing methods.

## Hypothesis

LV internal flow will be significantly increased in patients with dyssynchronous heart failure compared to healthy, age and sex-matched volunteers.

## Methods

**Figure 1:**
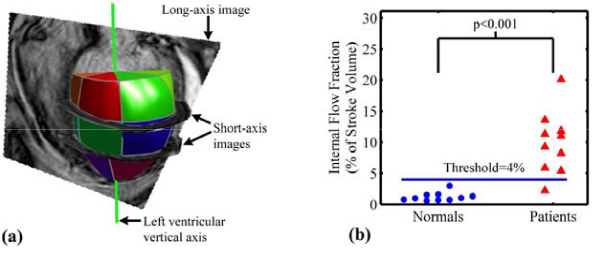
**(a) The 3-dimensional left ventricular volume is superimposed on the 4-chamber long-axis MRI**. Two of the short-axis images are also displayed to demonstrate the spatial arrangement of the images. The left ventricle was divided into 16 wedge-shaped regional volumes as shown. **(b)** Interal Flow Fraction (IFF) discriminates between patients and healthy controls with 95% accuracy.

Images were obtained with a 1.5 T Philips Intera scanner using a 5-element phased array cardiac coil. Short-axis steady-state free-precession (SSFP) cines were acquired over the length of the LV during breath-holds (8–10 mm slices with no gaps, 20 phases per cardiac cycle). Two and four-chamber long-axis cine images were also acquired. Ten patients with dyssynchronous heart failure (New York Heart Association class III/IV, LV ejection fraction < 35%, QRS > 150 ms) and 10 age and sex-matched healthy controls were imaged. The 3-dimensional LV volume was reconstructed and divided into 16 wedge-shaped volumes adjacent to the American Heart Association standardized myocardial segments (Fig 1A). Internal flow was defined as the sum of the magnitude of the volume changes in the 16 regions minus the magnitude of the global volume change over each time step in the cardiac cycle: *IF*(*t*) = ∑|Δ*V*(*t*)_*regional*_| - |Δ∑*V*(*t*)_*regional*_|. This difference is zero if no internal flow has occurred. Internal Flow Fraction (IFF) was defined as the total internal flow as a percentage of stroke volume.

## Results

**Figure 2:**
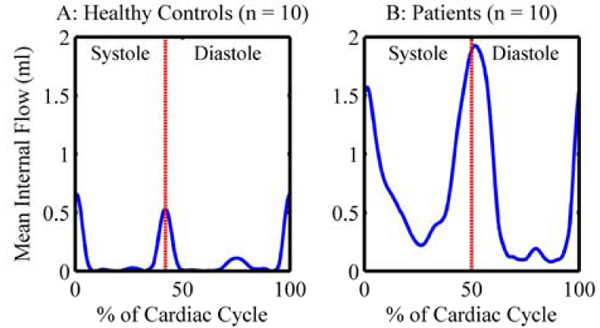
**Internal flow is significanly increased thoughout the cardiac cycle in patients with dyssycnhronous heart failure compared to age and sex-matched healthy controls**. Note the peaks in internal flow surrounding the periods of isovolumic contraction (time 0) and relaxation (vertical dashed line marking the end of systole).

IFF was significantly increased in the patients (10 ± 5% vs 1 ± 1% in the healthy controls, p < 0.001). An IFF threshold of 4% discriminated between patients and controls with 90% sensitivity and 100% specificity (Fig 1B). There were two large physiologic peaks of internal flow in the healthy controls: one during isovolumic contraction and another during isovolumic relaxation (Fig 2A). Internal flow occurred throughout the cardiac cycle in the patients, but peaked during the isovolumic periods (Fig 2B).

## Conclusion

Left ventricular Internal Flow Fraction can be quantified from images acquired in a standard cine cardiac MRI exam. Internal flow during the isovolumic periods is a normal, physiologic component of left ventricular contraction and relaxation. A left ventricular Internal Flow Fraction of 4% discriminated patients with dyssynchronous heart failure from age and sex-matched healthy controls with 95% accuracy.

